# Large-scale RNAi screen of G protein-coupled receptors involved in larval growth, molting and metamorphosis in the red flour beetle

**DOI:** 10.1186/1471-2164-12-388

**Published:** 2011-08-01

**Authors:** Hua Bai, Fang Zhu, Kapil Shah, Subba Reddy Palli

**Affiliations:** 1 Department of Entomology, S-225 Agriculture Science Bldg. N., University of Kentucky, Lexington, KY 40546, USA; 2 Department of Ecology and Evolutionary Biology, Brown University, Providence, RI 02912-G, USA

## Abstract

**Background:**

The G protein-coupled receptors (GPCRs) belong to the largest superfamily of integral cell membrane proteins and play crucial roles in physiological processes including behavior, development and reproduction. Because of their broad and diverse roles in cellular signaling, GPCRs are the therapeutic targets for many prescription drugs. However, there is no commercial pesticide targeting insect GPCRs. In this study, we employed functional genomics methods and used the red flour beetle, *Tribolium castaneum*, as a model system to study the physiological roles of GPCRs during the larval growth, molting and metamorphosis.

**Results:**

A total of 111 non-sensory GPCRs were identified in the *T. castaneum *genome. Thirty-nine of them were not reported previously. Large-scale RNA interference (RNAi) screen was used to study the function of all these GPCRs during immature stages. Double-stranded RNA (dsRNA)-mediated knockdown in the expression of genes coding for eight GPCRs caused severe developmental arrest and ecdysis failure (with more than 90% mortality after dsRNA injection). These GPCRs include dopamine-2 like receptor (TC007490/D2R) and latrophilin receptor (TC001872/Cirl). The majority of larvae injected with TC007490/D2R dsRNA died during larval stage prior to entering pupal stage, suggesting that this GPCR is essential for larval growth and development.

**Conclusions:**

The results from our study revealed the physiological roles of some GPCRs in *T. castaneum*. These findings could help in development of novel pesticides targeting these GPCRs.

## Background

G protein-coupled receptors (GPCRs) are seven-transmembrane receptor proteins that sense external signals and activate a variety of intercellular pathways. Binding of signal molecules causes the activation of the heterotrimeric G protein complex. GPCRs are members of the largest membrane receptor family, and the members of this family are present in almost all the eukaryotes [1]. Genome annotation efforts identified about 200 GPCRs in *Drosophila melanogaster *[2], 276 GPCRs in *Anopheles gambiae *[3], and 90 GPCRs in *Bombyx mori *[4]. Interestingly, a large number of GPCRs belong to olfactory and gustatory receptor classes [5]. So far about 60 olfactory receptors and 60 gustatory receptors were identified in *D. melanogaster *[6-8]. In the honey bee, *Apis mellifera*, genes coding for 35 neuropeptide receptors and 19 biogenic amine receptors were identified [9]. Right after the red flour beetle *Tribolium castaneum *genome sequences published in 2008 [10], 72 neurohormone GPCRs were identified from *Tribolium *genome, which includes 20 biogenic amine receptors, 48 neuropeptide receptors and four protein hormone receptors [11]. Since most of the identified *Tribolium *GPCRs in the above study belong to Class A Rhodopsin-like GPCR, a comprehensive annotation is required for a genome-wide functional analysis.

The large number and structural diversity among the members of GPCR gene family made it difficult to systematically study their biological functions. However, their diverse functions, such as the regulation of sense of vision, smell and taste, behavioral and mood regulation, immune system and autonomic nervous system, draw most attention in the pharmaceutical industry [12,13]. About 40% of therapeutic drugs target human GPCRs [14]. Insect development, reproduction, and various behaviors such as host finding and ecdysis, are also under the control of GPCRs [15]. For example, several GPCRs are known to participate in the regulation of insect ecdysis behavior. These include ecdysis triggering hormone receptor (ETHR), crustacean cardioactive peptide receptor (CcapR) and bursicon receptor (rk) [16-18]. Many of these GPCRs belong to biogenic amine and neuropeptide receptor families. Relatively few efforts have been made to apply the knowledge of insect GPCRs to the development of novel pest control methods such as the disruption of neuropeptide signaling systems using neuropeptide mimics [19,20]. Based on mode of action classification issued by Insecticide Resistance Action Committee (IRAC), there are no commercial synthetic pesticides available that use insect GPCR as targets http://www.irac-online.org. To discover GPCR pesticide targets, we identified 111 non-sensory GPCRs using BLAST search in the genome of the red flour beetle, *T. castaneum*. Thirty-nine of them are newly identified in this study. We then performed a large-scale RNAi screen to determine the function of all 111 identified *T. castaneum *GPCRs. RNAi for 25 GPCRs resulted in more than 30% mortality, including eight GPCRs with more than 90% mortality. Knockdown in the expression of the genes coding for these GPCRs severely affected larval growth, wing development and ecdysis. Finally, RNAi for one of the GPCRs, dopamine-2 like receptor (TC007490/D2R), resulted in high lethality of early larval growth suggesting TC007490 may play an essential role in larval growth and development.

## Results

### Identification of non-sensory GPCR genes in *T. castaneum*

Because olfactory and gustatory receptor families contain large number of receptors and are highly diverse multigene families, we excluded both chemo-sensory GPCRs as well as opsins in this study to reduce the amount of screen work. To identify non-sensory GPCRs in *T. castaneum *genome, amino acid sequences of known non-sensory GPCRs belonging to four different classes were retrieved from the GPCR database, GPCRDB http://www.gpcr.org/7tm/[21,22] and used as queries to perform BLASTP searches against *T. castaneum *protein database (Glean predictions) downloaded from Beetlebase http://beetlebase.org/ (Hereafter, we will refer 'non-sensory GPCR' as 'GPCR'). About 200 putative *T. castaneum *GPCRs with relative high homology to GPCRs of other species (E-values lower than 10-10) were selected for the next round of search. NCBI conserved domain search program http://www.ncbi.nlm.nih.gov/Structure/cdd/cdd.shtml was used to verify the presence of seven transmembrane domains (7TM) in all putative GPCRs. 111 GPCRs out of 200 hits were confirmed and classified into four different families: Class A, Rhodopsin-like receptor; Class B, Secretin receptor-like; Class C, Metabotropic glutamate receptor-like and Class D, Atypical GPCRs (see Additional file 1). Out of these 111 GPCRs, 72 of them were previously identified as biogenic amine and neuropeptide GPCRs [11]. We have classified them into above four classes based on conserved domain prediction program. The deduced amino acid sequences of the newly identified *T. castaneum *GPCRs were compared with that of *D. melanogaster *GPCRs. The closest homologues were identified based on the phylogenetic analysis and sequence homology search (see Additional files 1, 2, 3, 4 and 5).

As shown in Table 1, there are 74 Rhodopsin-like GPCRs, 19 Secretin receptor-like GPCRs, 11 Metabotropic glutamate receptor-like GPCRs, and 7 Atypical GPCRs. In Rhodopsin-like GPCR Class, there are 20 biogenic amine receptors (16 in *B. mori *and 22 in *D. melanogaster*), 42 peptide receptors (35 in *B. mori *and 38 in *D. melanogaster*), four glycoprotein hormone receptors (two in *B. mori *and four in *D. melanogaster*), one purine receptor (one in *B. mori *and one in *D. melanogaster*) were identified in *T. castaneum*. A comprehensive review on neurohormone GPCRs in *T. castaneum *by Hauser et al [11] is recommended for further reading. Recently, GRHR-like receptors TC009772 and TC001245 had been identified as adipokinetic hormone receptor (AKHR) [23] and AKH/corazonin-related peptide (ACP) receptor [24], respectively. Methuselah receptors belong to Class B GPCR and are involved in modulation of life span and stress response in *D. melanogaster *[25]. Only three methuselah receptors were identified in *T. castaneum *compared to 15 in *D. melanogaster *(see Additional files 1 and 3). In Class C, three Metabotropic GABAB receptors (same number as in *D. melanogaster*) and three metabotropic glutamate receptors (only one in *D. melanogaster *and one in *B. mori*) were identified in *T. castaneum *(see Additional files 1 and 4). The frizzled-like receptor belongs to Class D Atypical GPCRs. This group of receptors share less sequence conservation with the other GPCR families. In *T. castaneum *there are three frizzled-like receptor genes (four in *D. melanogaster *and two in *B. mori*), no ortholog for *D. melanogaster *frizzled 3 was found in *T. castaneum *genome (see Additional files 1 and 5).

**Table 1:** The number of *T. castaneum *GPCRs of each class in comparison to *B. mori *and *D. melanogaster*

	*T. castaneum*	*B. mori*	*D. melanogaster*
**Class A Rhodopsin-like**	**74**	**63**	**71**
Biogenic amine	20	16	22
Peptide	42	35	38
Protein hormone	4	2	4
Purine	1	1	1
Others	7	9	4

**Class B Secretin receptor-like**	**19**	**9**	**24**

**Class C Metabotropic glutamate receptor-like Metabotropic glutamate receptor-like**	**11**	**9**	**9**

**Class D Atypical GPCRs**	**7**	**3**	**6**

**Total**	**111**	**84**	**108**

Data for *T. castaneum *are from this study and [11]. Data for *B. mori *are from [4]and *D. melanogaster *are from [2,9] with modification.

### RNAi screen to identify GPCRs involved in larval and pupal development in *T. castaneum*

To study the role of each GPCR in regulating larval development in *T. castaneum*, dsRNA for 111 *T. castaneum *GPCRs were synthesized and injected into one-day-old final instar larvae. Mortality and development defects of dsRNA injected insects were recorded every 2-3 days until all adults eclosed. *Escherichia coli malE *gene that encodes maltose-binding protein has been used as an RNAi control in our lab for many years. It has been proved to be a reliable control since injection of dsRNA for *malE *gene doesn't affect *Tribolium *larval development, molting, growth and female reproduction. In the first RNAi screen, we found that out of 111 GPCR RNAi, knockdown in the expression of 18 GPCRs in Class A, five in Class B and two in Class D caused more than 30% of total mortality (see Additional file 6). No severe mortality was observed in insects injected with dsRNA for Class C GPCRs. To confirm this result, we repeated RNAi experiment by injecting dsRNA targeting identified 25 GPCRs. As shown in Table 2, we found that most of mortality results are repeatable, although there is some variation in stage-specific mortality. This variation may be due to synchronization issue of final instar larvae that were used for dsRNA injection, since it may be critical for some GPCRs to knock down their expression at the early stage of final instar larva. Among 25 GPCRs tested, there are genes coding for biogenic amine receptor (TC007490/D2R), peptide receptors (TC013945/CcapR, TC012493/ETHR, TC004716 and TC006805/CCHa2r), and protein hormone receptors (TC008163/bursicon receptor and TC009127/glycoprotein hormone-like receptor). Silencing of genes coding for four GPCRs (TC012521/stan, TC009370/mthl and TC001872/Cirl) in Class B and two GPCRs (TC014055/fz and TC005545/smo) in Class D also caused severe mortality (Table 2).

**Table 2:** Summary of RNAi for 25 GPCRs with high mortality phenotype in *T. castaneum*

			First Trial		Second Trial	
				
List #	Class	Official ID	Larva Mortality	Pupa Mortality	Larva Mortality	Pupa Mortality
/	/	*malE*	6.7%	0.0%	2.4%	2.4%
1	Class A	TC004470	12.5%	25.0%	13.3%	33.3%
2	Class A	TC007490	64.3%	35.7%	100.0%	0.0%
3	Class A	TC012447	11.1%	22.2%	18.8%	12.5%
12	Class A	TC011960	0.0%	71.4%	*	*
13	Class A	TC013979	11.1%	22.2%	25.0%	12.5%
14	Class A	TC011667	25.0%	25.0%	25.0%	0.0%
21	Class A	TC008163	10.0%	90.0%	21.2%	75.8%
22	Class A	TC009127	50.0%	16.7%	40.0%	0.0%
28	Class A	TC007170	45.5%	0.0%	26.7%	13.3%
30	Class A	TC006805	0.0%	62.5%	9.1%	54.5%
32	Class A	TC013945	0.0%	100.0%	42.1%	52.6%
33	Class A	TC012493	20.0%	60.0%	*	*
44	Class A	TC003150	0.0%	25.0%	7.1%	28.6%
61	Class A	TC004716	0.0%	41.7%	38.9%	22.2%
62	Class A	TC002068	33.3%	0.0%	33.3%	6.7%
66	Class A	TC014211	0.0%	30.0%	36.8%	5.3%
72	Class A	TC013650	9.1%	36.4%	22.2%	27.8%
73	Class A	TC015120	0.0%	33.3%	14.3%	28.6%
75	Class B	TC001222	10.0%	0.0%	12.5%	12.5%
81	Class B	TC001872	55.6%	44.4%	68.4%	31.6%
84	Class B	TC009370	0.0%	90.0%	42.9%	57.1%
90	Class B	TC010267	0.0%	40.0%	14.3%	35.7%
93	Class B	TC012521	0.0%	90.0%	31.6%	68.4%
105	Class D	TC014055	0.0%	100.0%	60.0%	40.0%
108	Class D	TC005545	0.0%	92.3%	46.7%	53.3%

Asterisk indicates that RNAi for two GPCRs (TC012493 and TC011960) were not carried out at the second trial.

**Figure 1:**
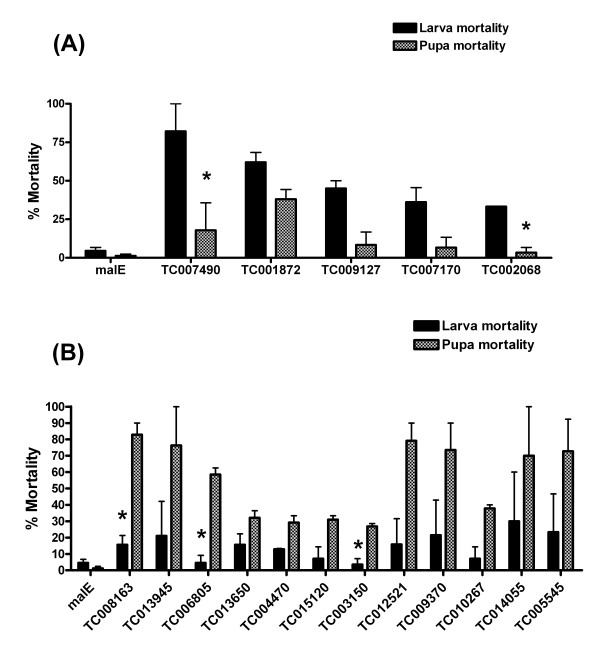
**Comparison of larva and pupa mortality caused by GPCR RNAi in *T. castaneum***. Stage-specific mortality pattern among different GPCR RNAi insects was compared. RNAi for five GPCRs results in most lethality occurred during the larval stage (Figure. 1A), while RNAi for 12 GPCRs caused relatively more pupal mortality than the larval mortality (Figure. 1B). dsRNA for *malE *(control) and selected *T. castaneum *GPCR were injected into one-day old final instar larvae. The percent mortality caused by dsRNA injection during the larval and pupal stages is shown. Mean ± SE of two independent experiments are shown.

We observed that there is a clear stage-specific mortality pattern among different GPCR RNAi insects. RNAi for five GPCRs results in most lethality occurred during the larval stage (Figure 1A), while RNAi for 12 GPCRs caused relatively more pupal mortality than the larval mortality (Figure 1B). This result suggests that each of the identified GPCR may play critical roles at different developmental stages.

Four genes were selected to check RNAi knockdown efficiency (see Additional file 7). They are associated with a range of mortality phenotype, e.g. TC007490 (100% total mortality), TC012521 (95% total mortality), TC006805 (75.6% total mortality), TC013650 (47.7% total mortality). About 50% or more knockdown efficiency was observed in dsRNA injected insects when compared to insects injected with control *malE *dsRNA for all four GPCRs tested. We also notice that knockdown efficiency is not reflecting how strong the phenotype is. For example, about 4-fold reduction on TC006805 transcripts caused by TC006805 RNAi results in 75.6% total mortality, while 2-fold reduction on TC012521 transcripts caused by RNAi results in 95% total mortality. Due to large amount of screen work, we didn't check RNAi knockdown efficiency for all GPCRs. Therefore, we cannot rule out the possibility that the absence of mortality phenotype is due to insufficient silencing of target GPCRs. In our studies where the expression of more than 500 genes was knocked down in *T. castaneum*, the lack of phenotype is rarely due to insufficient silencing [26-28]. RNAi works efficiently in *T. castaneum *and we observed more than 50% of silencing for more than 95% of the genes tested.

### Eight GPCRs are required for larval and pupal development

**Figure 2:**
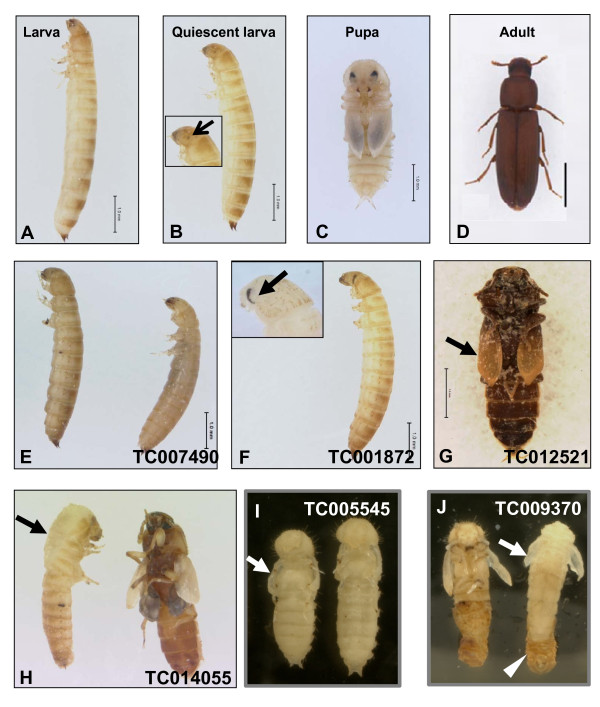
**Phenotypes observed after the injection of dsRNA for select GPCR into *T. castaneum***. dsRNA for *malE *(control) and selected *T. castaneum *GPCR were injected into one-day old final instar larvae. Phenotypes observed in control insects at three days after ecdysis to the final instar larval stage (A); quiescent stage, a non-feeding prepupal stage (B); three days after ecdysis to the pupal stage (C); three days after adult eclosion (D) are shown. (E). Phenotypes of TC007490/D2R RNAi insects. (F). Phenotypes of TC001872/Cirl RNAi insects. Accumulation of the ommatidia (black arrow) at quiescent stage is shown at the upper-left panel at higher magnification. (G). Phenotypes of TC012521/stan RNAi insects with wings attached to the ventral side of the abdomen (black arrow). (H). Phenotypes of TC014055/fz RNAi insects. The black arrow points to the split in the dorsal thoracic region. (I). Phenotypes of TC005545/smo RNAi insects with unextended pupal wings (white arrow). (J). Phenotypes of TC009370/mthl RNAi insects with improperly folded wings (white arrow) and unshed exuviae (white arrow head). Scale bar: 1.0 mm.

The phenotypes caused by silencing genes coding for six GPCRs, TC007490/D2R, TC001872/Cirl, TC012521/stan, TC009370/mthl, TC014055/fz and TC005545/smo were further investigated, since RNAi for these GPCRs results in more than 90% of total mortality. We recently reported characterization of TC008163/bursicon receptor RNAi phenotypes [29] and the study on TC013945/CcapR had been published previously [17]. Therefore, the roles of TC008163 and TC013945 on development are not included in the current study. The majority of insects injected with TC007490/D2R dsRNA died during the larval stage and was not able to molt to the pupal stage (Figure 2E). Only a few larvae injected with TC007490/D2R dsRNA were able to reach quiescent stage (a non-feeding prepupal stage, about 96 hr after ecdysis into final instar), suggesting that this gene may play an important role during larval growth and development rather than molting and metamorphosis. In contrast, most of the insects injected with TC001872/Cirl dsRNA entered the quiescent stage and died during this stage (Figure 2F). About 40% of the insects injected TC001872/Cirl dsRNA were able to molt to the pupal stage and eventually died during the early pupal stage. As shown in Figure 2F, the eyes continued to develop and accumulate the ommatidia during the quiescent stage in TC001872/Cirl RNAi insects, suggesting a premature eye development is triggered by TC001872/Cirl silencing.

The majority of insects injected with TC012521/stan dsRNA was not able to complete adult eclosion and died during pharate adult stage. The majority of TC012521/stan RNAi insects died from early eclosion arrest as their wings were not able to invert to the dorsal side as in control insects (Figure 2G). These data suggest that the pupal-adult ecdysis behavior was initiated but not completed in TC012521/stan RNAi insects.

Interestingly, TC014055/fz RNAi caused an arrest in both larval-pupal and pupal-adult ecdysis, suggesting that TC014055/fz may play important roles in the regulation of ecdysis behavior. As shown in Figure 2H, a split in the dorsal thoracic region was seen in the pharate pupae from TC014055/fz RNAi insects, indicating that larval-pupal ecdysis began, but the shedding of the larval cuticle was not completed. During the pupal-adult ecdysis, the phenotypes of TC014055/fz RNAi insects are similar to that of TC012521/stan RNAi with the wings still attached to the ventral side of the abdomen. The majority of insects injected with TC005545/smo dsRNA died during the early pupal stages without showing any ecdysis defects. However, unlike in the control insects injected with *malE *dsRNA (Figure 2C), the pupal wings were not fully extended in TC005545/smo RNAi insects (Figure 2I). Insects injected with TC009370/mthl dsRNA were arrested at the late phase of larval-pupal and pupal-adult ecdysis. Most of the body parts of TC009370/mthl RNAi insects were able to escape from the old cuticle, however, the wings were not able to fold properly and the exuviae was still attached to the abdomen (Figure 2J).

### Expression pattern of GPCRs during the larval and pupal stages

**Figure 3:**
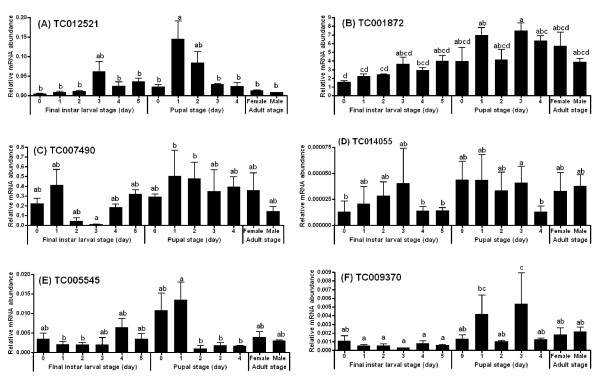
**Stage-specific gene expression of six GPCRs in the whole body determined by qRT-PCR**. Total RNA was extracted from pools of five larvae, pupae, or adult (three day-old males and females) for each time point. The Y-axis denotes expression levels normalized using *Tcrp49 *mRNA levels as an internal control. Mean ± SE of three replications are shown. Means with the same letter are not significantly different (α = 0.05; ANOVA).

Real-time qRT-PCR was used to study the stage-specific expression pattern of six identified GPCRs that are involved in larval development and metamorphosis. The mRNA levels of TC012521/stan showed two peaks during larval and pupal stages, one small peak at three-day after ecdysis to the final instar larvae stage and one large peak around one-day after ecdysis to the pupal stage (Figure 3A). The mRNA levels of TC001872/Cirl were the highest among seven GPCRs tested and the mRNA levels during the pupal stage were higher relative to that in the larval stage (Figure 3B). The mRNA levels of TC007490/D2R were extremely lower in the middle of final instar larval stage when compared to early and late final instar larval stages. The mRNA levels of TC007490/D2R remained high during the pupal stage (Figure 3C). The mRNA levels of TC014055/fz were the lowest among the 7 GPCRs tested (Figure 3D). Interestingly, TC005545/smo showed higher mRNA levels during the larval-pupal transition with a peak at one-day after ecdysis to the pupal stage (Figure 3E). The mRNA levels of TC009370/mthl were also relatively low and two small peaks were detected at one-day and three-day after ecdysis to the pupal stage (Figure 3F). Thus, the stage-specific expression pattern of these GPCRs may suggest that they play specific roles in the regulation of larval growth as well as larval-pupal and pupal-adult transition.

## Discussion

Because of their diverse biological functions activated by a wide range of ligands, GPCRs have been the targets for a majority of therapeutic drugs. However, to date, no commercial insecticides have been developed that target insect GPCRs http://www.irac-online.org. Our large-scale RNAi screen for 111 annotated GPCRs in *T. castaneum *identified 18 genes in Class A, five genes in Class B and two genes in Class D whose RNAi resulted in more than 30% larvae and pupae mortality, suggesting they may play important roles in larval growth, development and metamorphosis. Among these genes, silencing of six of them (TC007490/D2R, TC001872/Cirl, TC012521/stan, TC009370/mthl, TC014055/fz and TC005545/smo) caused more than 90% mortality during the final instar larval and pupal stages. A majority of the insects injected with dsRNA for TC007490/D2R died as larvae. These data suggest that TC007490/D2R is one of the essential GPCRs for normal larval growth and development. Therefore, TC007490/D2R is one of the potential targets for the development of novel pesticides for controlling the red flour beetle and related insect pests.

Unlike the dopamine 1-like receptor, D2R transduces second-messenger signaling via inhibitory G proteins (Gαi/o), which leads to inhibition of adenylate cyclase and modulation of ion channels [30]. D2R RNAi flies showed decreased locomotor activity [31]. In *D. melanogaster*, D2R is one of the highly expressed genes in the head and brain http://www.flyatlas.org/ and D2R RNAi in a primary neuron culture caused an excessive branching phenotype (36), suggesting its important role in early neuronal development and central nerve system function. Since TC007490/D2R RNAi beetles died during the larval stage, TC007490/D2R might be playing a critical role in the growth and development of larvae perhaps by modulating neuronal development and locomotor activity as reported in *D. melanogaster*. However, further research is needed to determine the precise mechanism of TC007490/D2R function in the regulation of growth and development of *T. castaneum *larvae. The biological function for Cirl, the other GPCR identified in our RNAi screen as that required for larval growth and development, is not known. However, in vertebrates this receptor is activated by a neurotoxin found in the venom of the black widow spider [32]. TC001872/Cirl could be another interesting target for development of novel pesticides.

In contrast to TC007490/D2R RNAi, three other GPCR RNAi results significant pupal lethality, rather than larval lethality. They are TC008163/bursicon receptor, TC006805/CCHa2r and TC003150/sNPF-R. Recently, the role of bursicon receptor (rk) in larval development was identified in *D. melanogaster *[18]. When *D. melanogaster *rk expression was down-regulated by transgenic RNAi, the majority of progeny died at pupal stage. Similar to our previous study [30], rk expression is required in developing epidermal tissue and imaginal discs in *D. melanogaster *[18]. Early this year, two *Drosophila *GPCRs code for novel receptors for CCHamide-1 and -2 were deorphanized [31]. In *B. mori*, one of the CCHamide receptor genes is expressed in central nervous system and gut, however, the biological function for this neuropeptide receptor is unknown [32]. In the present study, we found knockdown of one of the putative CCHamide receptors (TC006805/CCHa2r) in *T. castaneum *results in relative high mortality in pupal stage, indicating that this novel neuropeptide receptor may play a role during the metamorphosis. Further studies are needed to investigate the specific function of the CCHamide receptor during larval or pupal development. In *Drosophila*, short neuropeptide F (sNPF) and its receptor sNPFR1 regulate expression of insulin-like peptide in brain IPC neurons [33]. Therefore the biological function of sNPF has been linked to growth, metabolism and aging. Although we didn't find a huge mortality by knocking down TC003150/sNPF-R, RNAi for TC003150 caused higher pupal lethality than larval lethality, suggesting its critical role in pupal development, probably imaginal disc growth.

Among eight GPCR RNAi that result severe mortality, insects injected with TcCcapR2 dsRNA were not able to complete adult eclosion and died during the pharate adult stage. Our data showed that the pupal-adult ecdysis behavior was initiated but not completed in TcCcapR2 RNAi insects. In *D. melanogaster*, Ccap-cell ablation resulted in deficiencies in both pupal and adult ecdysis [33,34]. Similarly, a recent study on Ccap and its receptors in *T. castaneum *showed that Ccap and CcapR2 RNAi caused the ecdysis deficiency associated with weak reverse-bending, wing air-filling and anterior-posterior (A-P) contractions [17]. In addition, TC012521/stan, a cadherin-related GPCR involved in tissue polarity, was also found to play a role in pupal-adult ecdysis. The *D. melanogaster *tissue polarity gene *stan *(starry night) possesses a huge protocadherin domain containing nine cadherin motifs, four EGF-like motifs, and two laminin G motifs. Flies with *stan *mutant in the wing show cell autonomous wing hair polarity defects [35], while *stan *mutant in the eye show the tissue polarity defects in the ommatidia [36]. The dependence of proper *stan *localization on Frizzled (Fz) activity suggests that *stan *functions downstream of Fz in controlling planar polarity [37]. Interestingly, RNAi for Frizzled in *T. castaneum *caused the arrest in both larval-pupal and pupal-adult ecdysis. A split in the dorsal thoracic region was seen in the pharate pupae, indicating larval-pupal ecdysis began but pupae were not able to come out of the larval cuticle. Frizzled is a wingless receptor and a tissue polarity gene. In *D. melanogaster*, frizzled mutants show strong wing-hair disorientation, negligible segment-polarity of homozygous embryos, altered polarity of bristles and hairs on the adult abdomen and thorax [38,39]. Silencing of another tissue polarity gene, TC005545/smo, did not result in any arrest of ecdysis, but caused defects in wing expansion. Smoothened (*smo*) is thought to be a component of the Hedgehog receptor. *Smo *mutation caused embryonic lethality. In *smo *mutants, all denticles in abdominal segments point posterior. *Smo *is also required for *decapentaplegic *(*dpp*) expression in wing discs during the metamorphosis [40]. Taken together, these data suggest that smo regulates wing development during the postembryonic stages in a conserved manner across insect species. These results also indicate that the tissue polarity-regulating GPCRs play critical roles during molting and metamorphosis in *T. castaneum*.

Interestingly, one of the aging-related GPCRs, TC009370/mthl, was found to be involved in both larval-pupal and pupal-adult ecdysis. The Methuselah-like receptors (*mthl*) play an important role in aging and reproduction. Mutation of *mthl *resulted from a P-element insertion in *D. melanogaster *extended the life span and enhanced the resistance to free radicals and starvation [25]. Flies homozygous for the *mthl *mutation showed pre-adult lethality. However, there is no phenotypic data for the Methuselah-like 5 receptor (*mthl5*) mutant fly. In this study, we found that out of three Methuselah-like receptors, only TC009370/mthl regulates larval-pupal and pupal-adult ecdysis. This is similar to the pre-adult lethality observed in *D. melanogaster mthl *mutants.

Here we hope to identify GPCRs that could be served as potential pesticide targets, which can be used in small molecule screen, or the development of RNAi-based pesticides. Among the identified GPCRs, many of them belong to classic GPCRs, e.g. biogenic amine receptors (TC007490/D2R and TC011960/5-HTR) and neuropeptide receptors (TC009127/glycoprotein hormone-like receptor). These GPCRs can be used as potential targets for novel pesticide development. On the other hand, it may not be possible to apply small molecule ligands for pest management through targeting identified atypical GPCRs (e.g. TC014055/fz and TC005545/smo), however, it is possible to develop a RNAi-based pest control strategy through ingestion of specific dsRNA targeting atypical GPCRs as well as classical GPCRs [29].

## Conclusions

Large-scale RNAi screen has been successfully used in dissecting signal transduction networks *in vitro *[41,42], or biological processes *in vivo *[43]. In this study, we performed a non-sensory GPCR screen and identified at least six GPCRs that are required for either larval development or ecdysis behavior. Our study provides a systematic functional analysis on GPCRs in *T. castaneum *and increases our understanding on the function of insect GPCR in the regulation of post-embryonic development. In addition the identified GPCR genes could be potential pesticide targets for development of novel insecticides. Insecticide resistance has become a serious problem in controlling many insect pests that damage crops and transmit diseases. In Africa, the malaria vector, *A. gambiae *shows increasing levels of resistance to pyrethroid insecticides resulting from knock-down resistance (*kdr*) mutation [44] and overexpressed cytochrome P450 [45], which has significant impact on malaria prevention and control. Alternative control agents have to be developed to manage populations of insecticide-resistant pest insects. Since the GPCR receptor family has been successfully used as targets of many therapeutic drugs, it may be possible to develop pesticides that target GPCRs. The identified GPCRs in our study could be used as targets for development of novel pesticides.

## Methods

### Beetle rearing and staging

Strain GA-1 of *T. castaneum *was reared on organic wheat flour containing 10% yeast at 30°C under standard conditions [46]. The final instar larvae were identified as soon as they molted using untanned white cuticle [47]. The 4th instar larvae were identified based on the head capsule width (about 300-400 μm).

### Double-stranded RNA (dsRNA) synthesis

Primers containing GPCR gene sequences and T7 polymerase promoter (TAATACGACTCACTATAGGG) at the 5'-end of both the forward primer and reverse primer were used to amplify the 200-600 bp regions (Primer sequences available upon request). The PCR product was used as a template for dsRNA synthesis using the Ambion MEGAscript transcription kit (Ambion, Austin, TX). dsRNA was purified using a Phenol/chloroform extraction followed by ethanol precipitation and dissolved in nuclease-free water to a concentration of 3-5 μg/μl. The quality of dsRNA was checked by running on an agarose gel and the concentration was measured using NanoDrop1000 spectrophotometer (Thermo Fisher Scientific Inc., Waltham, MA).

### dsRNA Microinjection

Larvae were anaesthetized using ether vapors for 5 min, and then placed on double-sided sticky tape over a glass slide. 50-100 nl of dsRNA was injected into a larva on the lateral side of the second or third abdominal segments using an aspirator tube assembly (Sigma) fitted with a 3.5 inch glass capillary tube (Drummond) pulled by a needle puller (Model P-2000, Sutter Instruments Co., Novato, CA). Injected larvae were allowed to recover for 1 hour at room temperature, and then transferred to 30°C incubator. Control larvae were injected with dsRNA for *E. coli malE *gene. dsRNA for *E. coli malE *gene was amplified using LITMUS 28iMal control plasmid (New England Biolabs, Ipswich, MA) as a template. Mortality and development defects of dsRNA injected insects were recorded every 2-3 days until all adults eclosed.

### Imaging and documentation

RNAi phenotypes were photographed using the modular zoom system (Leica Z16 APO, Germany) fitted with JVC 3CCD Digital Camera KY-F75U. The images were documented using Cartograph version 6.1.0 (GT Vision Demonstration) and processed using Archimed version 5.2.2 (Micovision Instruments).

### cDNA synthesis and quantitative real-time reverse-transcriptase polymerase chain reaction (qRT-PCR)

Total RNA was extracted from the whole bodies of staged larvae and pupae using TRI reagent (Molecular Research Center Inc., Cincinnati, OH). cDNA synthesis by reverse transcription was performed using 2 μg of DNase I treated RNA and iScript cDNA synthesis kit (Biorad Laboratories, Hercules, CA) in a 20 μl reaction volume. qRT-PCR was performed using MyiQ single color real-time PCR detection system (Biorad Laboratories, Hercules, CA) in a 20 μl total reaction volume. Both the PCR efficiency and correlation coefficient were taken into account prior to estimating the relative gene expression. To determinate the stable housekeeping gene, the expression of five reference genes were examined across 72 cDNA samples collected from different developmental stages. Using normalization software package *Bestkeeper *[48], ribosomal protein rpL32/rp49 (GenBank accession no. XM_964471) has been found to be the least-variable reference gene (see Additional file 8 for more detail). Therefore all gene expression levels were normalized to rp49 by a standard curve based method [49,50]. Mean and standard errors for each time point were obtained from the averages of three independent biological replicates. Primer sequences are available upon request.

### Phylogenetic tree building

Full length GPCR protein sequences of the *T. castaneum *and one closest homologue for each GPCR from *D. melanogaster *were aligned in ClustalW. From these alignments, a phylogenetic tree was constructed using MEGA 4.0 program [51], according to the neighbor-joining method with a bootstrap test calculated with 1000 replicates and a poisson correction model.

### Statistical analysis

Student's t-Test and analysis of variance was performed using JMP 8.0 (SAS Institute Inc., Cary, NC) to test for statistical differences among treatments (α = 0.05). Pair-wise comparisons were made using Tukey-Kramer HSD method.

## Authors' contributions

HB and SRP designed research. HB, FZ and KS performed experiments and analyzed data. HB and SRP wrote the paper. All authors read and approved the final manuscript.

## Supplementary Material

Additional file 1 **List of identified *Tribolium *GPCR**. 111 non-sensory *Tribolium *GPCRs and their closest *Drosophila *homologs are grouped into four classes. Official ID from BeetleBase http://beetlebase.org is used for each *Tribolium *GPCR. Known or putative ligand for each *Tribolium *GPCR is based on previous publications. CG number, symbol and full name for *Drosophila *GPCRs are based on Flybase http://flybase.org/ with modification. Click here for file

Additional file 2 **Phylogenetic tree analysis of Class A Rhodopsin-like GPCRs from *T. castaneum *and *D. melanogaster***. The tree is rooted by *D. melanogaster *metabotropic glutamate receptor (CG11144). *Tribolium *and *Drosophila *GPCRs are indicated by official ID or CG number followed by putative ligands in parentheses. Click here for file

Additional file 3 **Phylogenetic tree analysis of Class B Secretin receptor-like GPCRs from *T. castaneum *and *D. melanogaster***. The tree is rooted by *D. melanogaster *FMRFamide receptor (CG2114). *Tribolium *and *Drosophila *GPCRs are indicated by official ID or CG number followed by putative ligands in parentheses. Gene symbol of methuselah-like GPCRs, Cirl and stan are also shown. Click here for file

Additional file 4 **Phylogenetic tree analysis of Class C Metabotropic glutamate receptor-like GPCRs from *T. castaneum *and *D. melanogaster***. The tree is rooted by *D. melanogaster *FMRFamide receptor (CG2114). *Tribolium *and *Drosophila *GPCRs are indicated by official ID or CG number followed by putative ligands in parentheses. Click here for file

Additional file 5 **Phylogenetic tree analysis of Class D Atypical GPCRs from *T. castaneum *and *D. melanogaster***. The tree is rooted by *D. melanogaster *FMRFamide receptor (CG2114). *Tribolium *and *Drosophila *GPCRs are indicated by official ID or CG number followed by putative ligands in parentheses. Gene symbol of frizzled GPCRs are also shown. Click here for file

Additional file 6 **Summary of mortality phenotype caused by RNAi targeting *Tribolium *GPCRs**. Larva, pupa and adult mortality resulted from *Tribolium *GPCR RNAi are shown. *E. coli malE *gene is used as a negative control. Asterisk indicates the mortality is calculated as the average of two trials. Click here for file

Additional file 7 **Knockdown efficiency of four selected GPCR RNAi during larval stage of *T. castaneum***. Total RNA was extracted from pools of five larvae (at quiescent stage) injected with *malE *or GPCR dsRNA. The Y-axis denotes relative expression levels normalized using *Tcrp49 *mRNA levels as an internal control. Mean ± SE of three replications are shown. Asterisk indicates a statistically significant difference between control and GPCR RNAi insects. Click here for file

Additional file 8 **Selection of stable reference gene using *BestKeeper***. To determinate the stable housekeeping gene, the expression of five reference genes were examined across 72 cDNA samples collected from different developmental stages (Larva day 1 to day 5, Quiescent larva day 1 and day 2, Pupa day 0 to day 6, Adult day 0 to day 2). (A). Based on the two most important criteria for evaluating the stability of reference genes by *Bestkeeper *program [48], the stability (SD value) and the relation to the *BestKeeper *index (r and P-value), five reference genes, Actin, Elongation factor 1-α (Ef1α), Ribosomal protein 49 (Rp49), Ribosomal protein s3 (Rps3), and Tubulin alpha 6 (Tubulinα6) are all stable in different developmental stages of *T. castaneum*. From the analysis, *rp49 *was chosen as the reference gene to calculate relative expression levels because it showed the most stable expression among samples. (B). Stable expression of five reference genes, Actin, Ef1α, Rp49, Rps3, and Tubulinα6 are shown across 12 RNA samples isolated from different developmental stages (LD1: Larva day 1; Q1: Quiescent larva day 1; PD0: Pupa day 0; AD0: Adult day 0). Products obtained after 40 cycles of PCR amplification under conditions described in the Materials and Methods section were resolved on an agarose gel. Click here for file
