# Supplementary figures and images for: Large-scale RNAi screen of G protein-coupled receptors involved in larval growth, molting and metamorphosis in the red flour beetle
Source: BMC Genomics. 2011 Aug 1;12:388. doi: 10.1186/1471-2164-12-388 (PMC3163568; doi:10.1186/1471-2164-12-388)

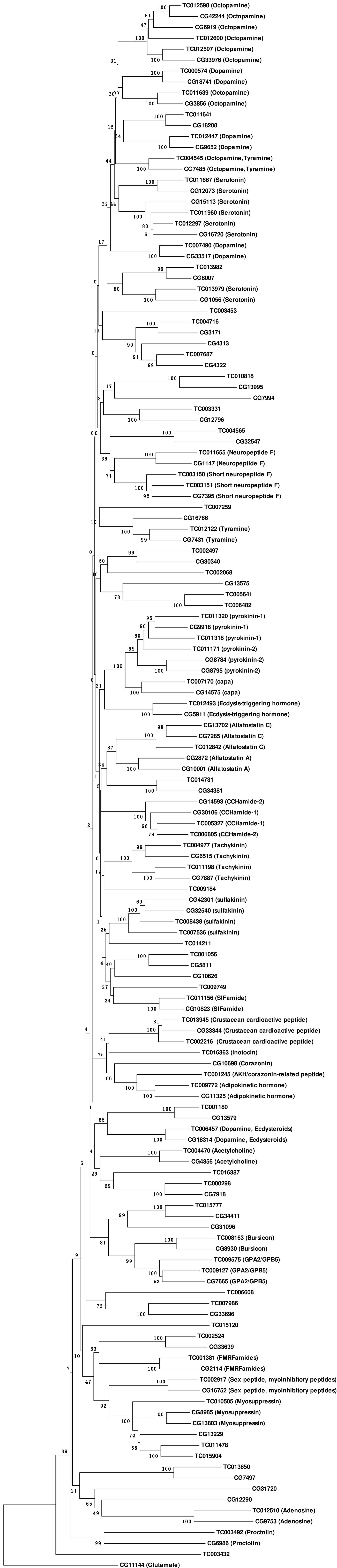

0.2

Supplement: Additional file 2 — Phylogenetic tree analysis of Class A Rhodopsin-like GPCRs from T. castaneum and D. melanogaster. The tree is rooted by D. melanogaster metabotropic glutamate receptor (CG11144). Tribolium and Drosophila GPCRs are indicated by official ID or CG number followed by putative ligands in parentheses. [file 1471-2164-12-388-S2.PDF]

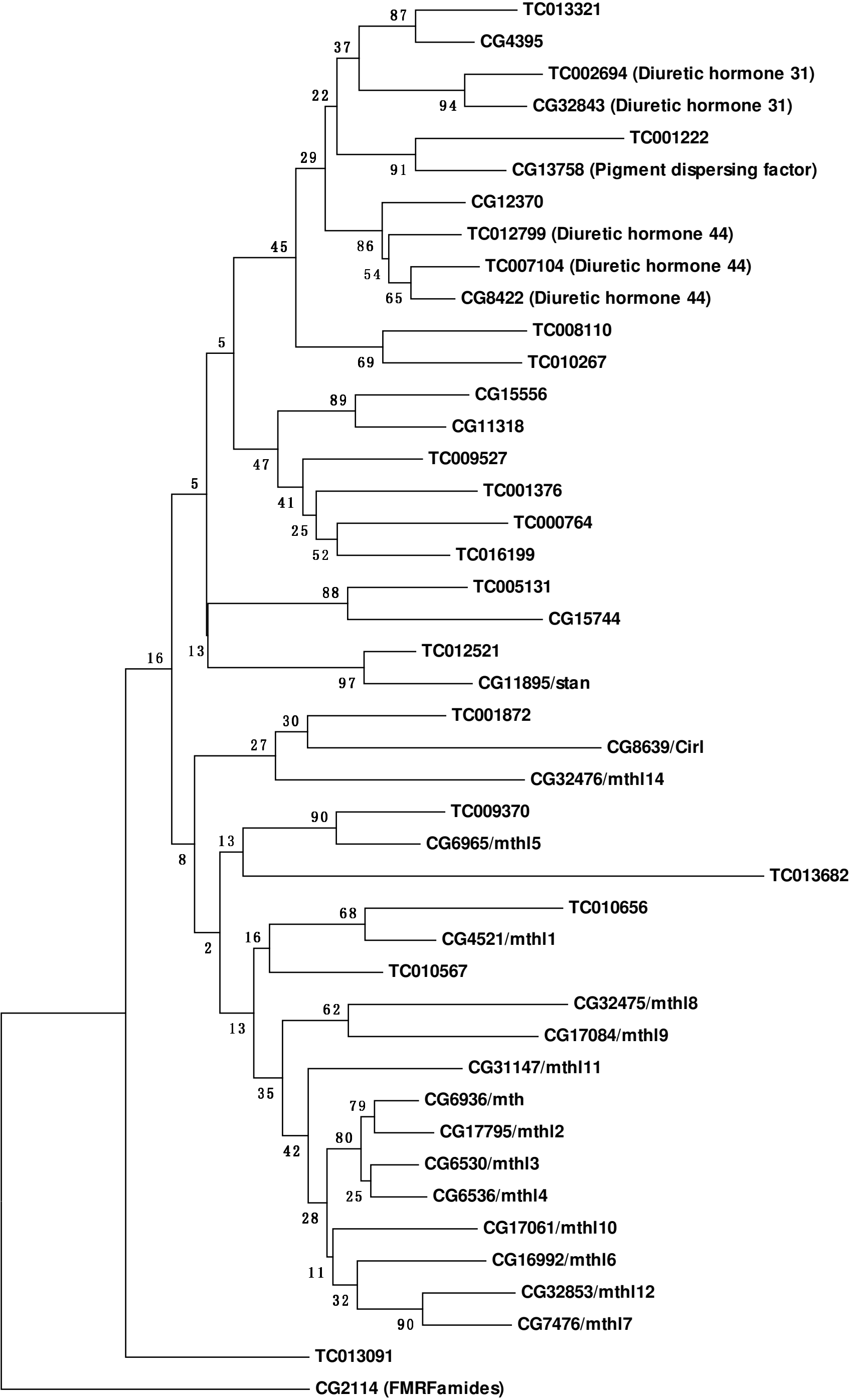

0.2

Supplement: Additional file 3 — Phylogenetic tree analysis of Class B Secretin receptor-like GPCRs from T. castaneum and D. melanogaster. The tree is rooted by D. melanogaster FMRFamide receptor (CG2114). Tribolium and Drosophila GPCRs are indicated by official ID or CG number followed by putative ligands in parentheses. Gene symbol of methuselah-like GPCRs, Cirl and stan are also shown. [file 1471-2164-12-388-S3.PDF]

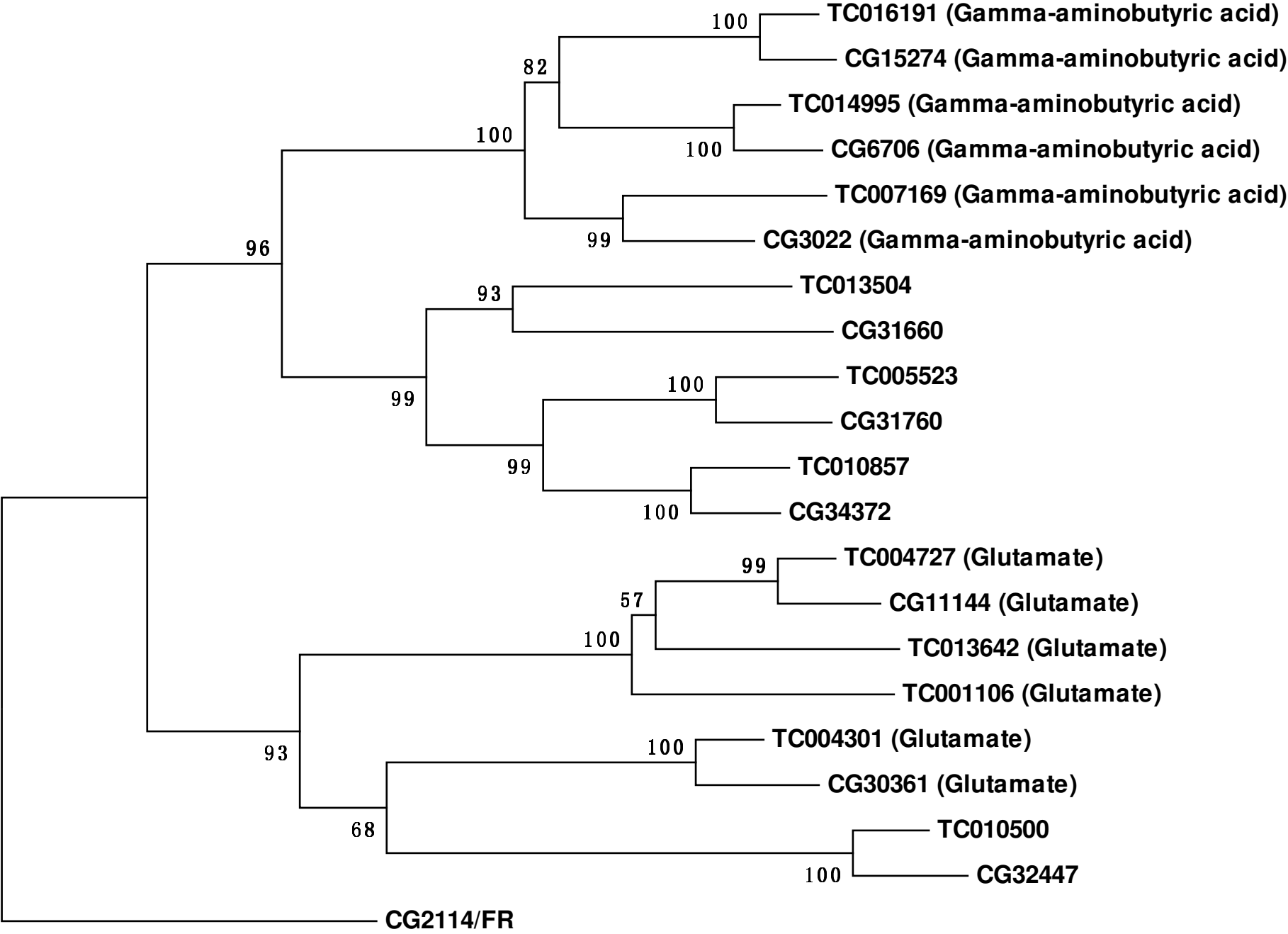

0.2

Supplement: Additional file 4 — Phylogenetic tree analysis of Class C Metabotropic glutamate receptor-like GPCRs from T. castaneum and D. melanogaster. The tree is rooted by D. melanogaster FMRFamide receptor (CG2114). Tribolium and Drosophila GPCRs are indicated by official ID or CG number followed by putative ligands in parentheses. [file 1471-2164-12-388-S4.PDF]

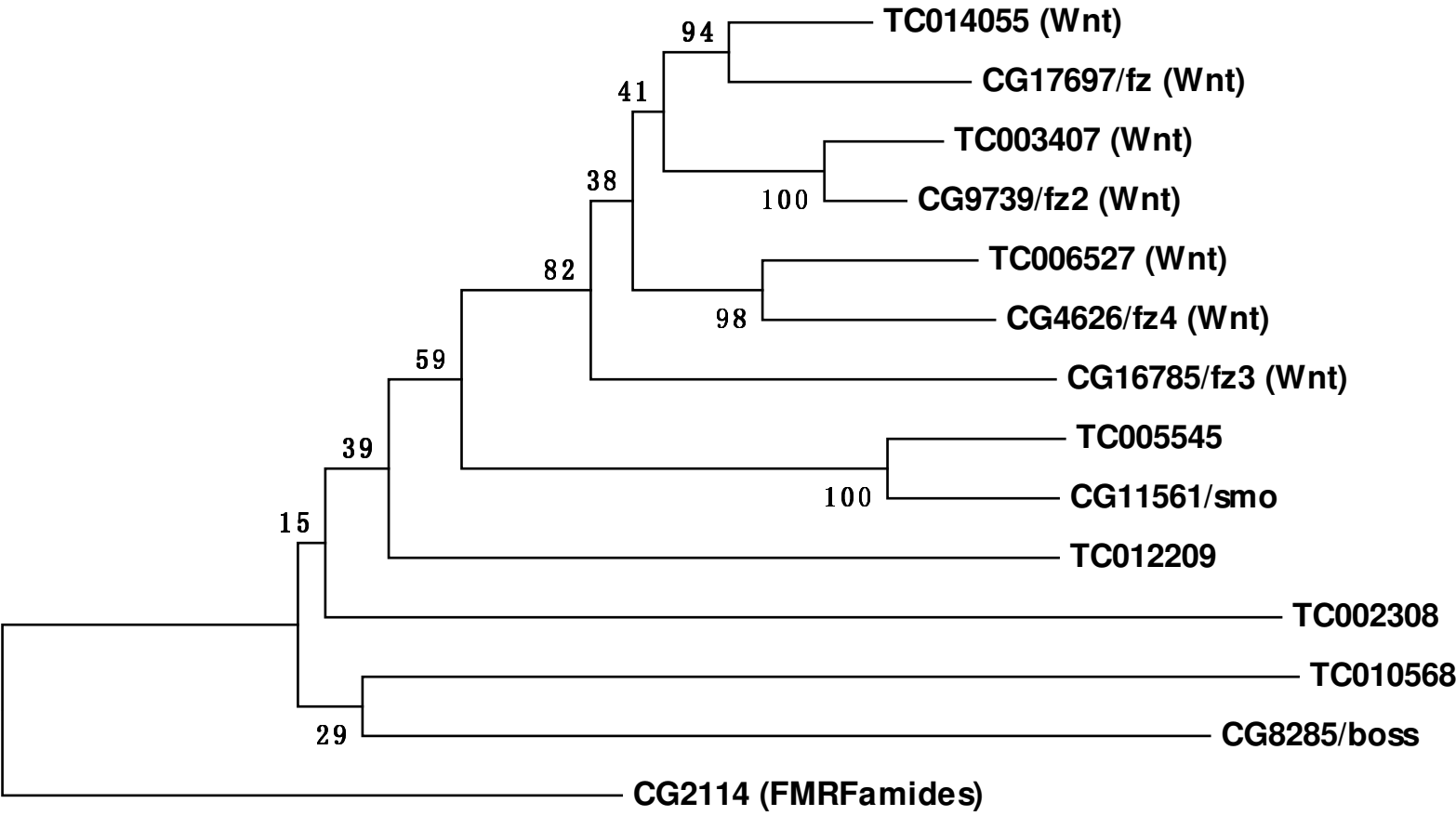

0.2

Supplement: Additional file 5 — Phylogenetic tree analysis of Class D Atypical GPCRs from T. castaneum and D. melanogaster. The tree is rooted by D. melanogaster FMRFamide receptor (CG2114). Tribolium and Drosophila GPCRs are indicated by official ID or CG number followed by putative ligands in parentheses. Gene symbol of frizzled GPCRs are also shown. [file 1471-2164-12-388-S5.PDF]

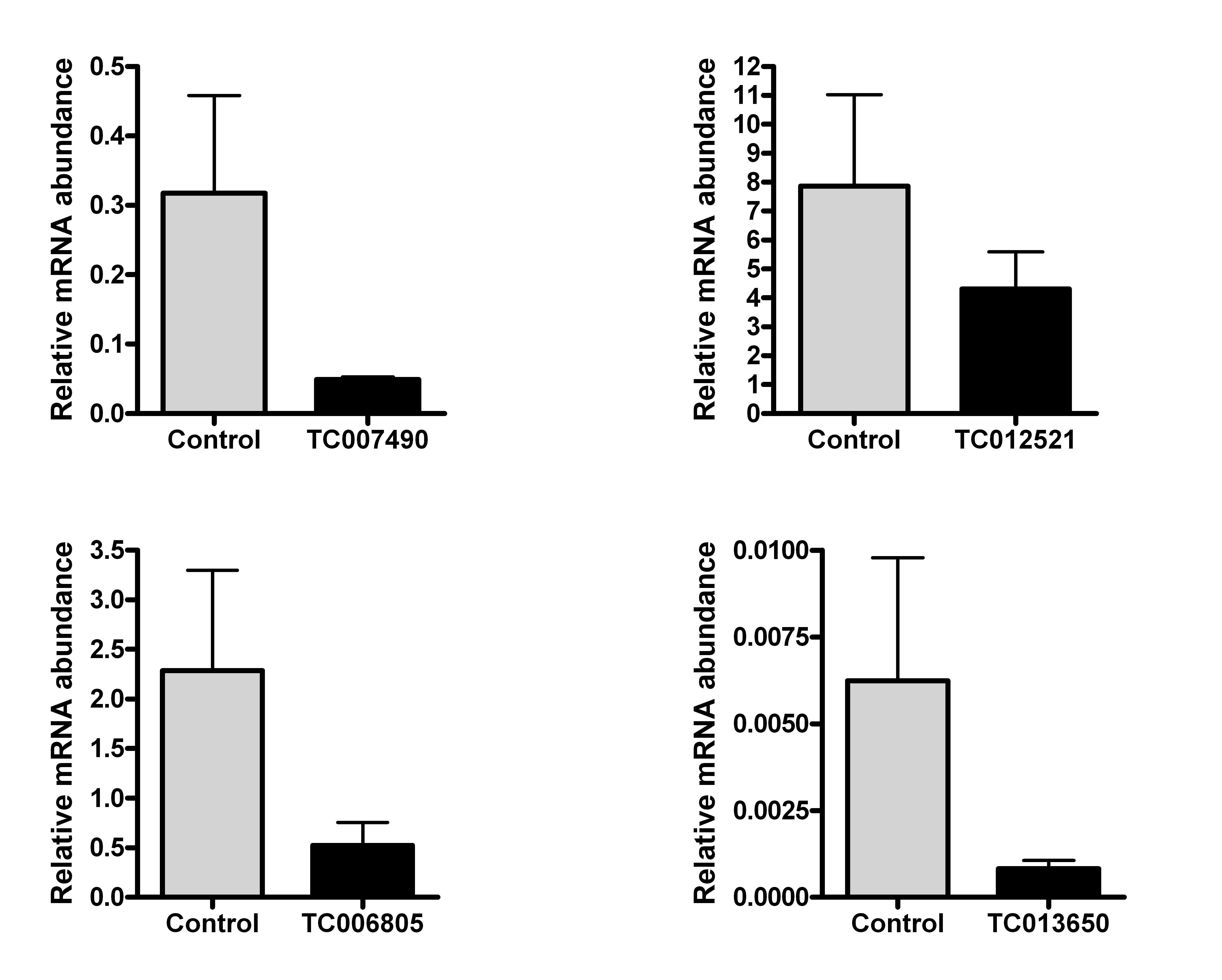

Supplement: Additional file 7 — Knockdown efficiency of four selected GPCR RNAi during larval stage of T. castaneum. Total RNA was extracted from pools of five larvae (at quiescent stage) injected with malE or GPCR dsRNA. The Y-axis denotes relative expression levels normalized using Tcrp49 mRNA levels as an internal control. Mean ± SE of three replications are shown. Asterisk indicates a statistically significant difference between control and GPCR RNAi insects. [file 1471-2164-12-388-S7.JPEG]
