# Supplementary material for: Large-scale RNAi screen of G protein-coupled receptors involved in larval growth, molting and metamorphosis in the red flour beetle
Source: BMC Genomics. 2011 Aug 1;12:388. doi: 10.1186/1471-2164-12-388 (PMC3163568; doi:10.1186/1471-2164-12-388)

(A). Statistical analyses of five candidate reference genes based on their threshold cycle (C_T) value

BestKeeper analyses of candidate reference genes (n=5)						
Parameter	*Actin*	*Ef1α*	*Rp49*	*Rps3*	*Tubulinα6*	*BestKeeper*
N	72	72	72	72	72	72
GM [C_T]	26.25	16.32	16.96	17.28	24.98	21.51
AM [C_T]	26.27	16.34	16.97	17.29	25.13	21.52
Min [C_T]	24.62	14.52	15.54	15.72	19.79	20.06
Max [C_T]	28.44	18.61	18.61	18.81	29.89	23.21
SD [± C_T]	0.81	0.65	0.55	0.55	2.38	0.53
CV [% C_T]	3.09	3.97	3.24	3.18	9.46	2.46
Pair-wise correlation analyses						
BestKeeper vs	*Actin*	*Ef1α*	*Rp49*	*Rps3*	*Tubulinα6*	
Coeff. of corr. [*r*]	0.784	0.649	0.825	0.786	0.765	
Coeff. of det. [r^2]	0.615	0.421	0.681	0.618	0.585	
P-value	0.001	0.001	0.001	0.001	0.001	

Abbreviations for listed parameters: n: number of candidate reference genes; N: sample size for each candidate reference genes as well as the *BestKeeper*; GM [C_T]: geometric means of the threshold cycle (C_T); AM [C_T]: the arithmetic mean of C_T; Min [C_T] and Max [C_T]: the extreme values of C_T; SD [±C_T]: the standard deviation of the C_T; CV [% C_T]: the coefficient of variance expressed as a percentage at the C_T level; The correlation between each candidate reference gene and *BestKeeper* index is calculated by the Pearson correlation coefficient [*r*], coefficient of determination [r^2], and the *P*-value.

(B). Stable expression profile of five reference genes

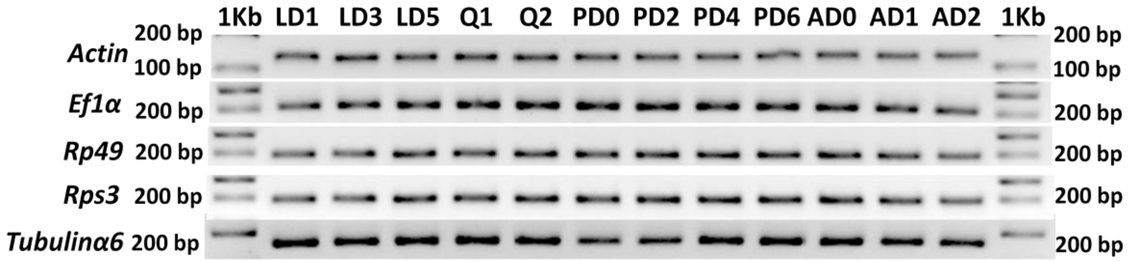

Supplement: Additional file 8 — Selection of stable reference gene using BestKeeper. To determinate the stable housekeeping gene, the expression of five reference genes were examined across 72 cDNA samples collected from different developmental stages (Larva day 1 to day 5, Quiescent larva day 1 and day 2, Pupa day 0 to day 6, Adult day 0 to day 2). (A). Based on the two most important criteria for evaluating the stability of reference genes by Bestkeeper program [48], the stability (SD value) and the relation to the BestKeeper index (r and P-value), five reference genes, Actin, Elongation factor 1-α (Ef1α), Ribosomal protein 49 (Rp49), Ribosomal protein s3 (Rps3), and Tubulin alpha 6 (Tubulinα6) are all stable in different developmental stages of T. castaneum. From the analysis, rp49 was chosen as the reference gene to calculate relative expression levels because it showed the most stable expression among samples. (B). Stable expression of five reference genes, Actin, Ef1α, Rp49, Rps3, and Tubulinα6 are shown across 12 RNA samples isolated from different developmental stages (LD1: Larva day 1; Q1: Quiescent larva day 1; PD0: Pupa day 0; AD0: Adult day 0). Products obtained after 40 cycles of PCR amplification under conditions described in the Materials and Methods section were resolved on an agarose gel. [file 1471-2164-12-388-S8.PDF]
